# Photosynthetic mechanisms underlying NaCl-induced salinity tolerance in rice (*Oryza sativa*)

**DOI:** 10.1186/s12870-024-04723-3

**Published:** 2024-01-10

**Authors:** Guanqiang Zuo, Jingxin Huo, Xiaohui Yang, Wanqi Mei, Rui Zhang, Aaqil Khan, Naijie Feng, Dianfeng Zheng

**Affiliations:** 1 College of Coastal Agricultural Sciences, Guangdong Ocean University, Zhanjiang, 524008 China; 2 College of Natural Resources and Environment, Northwest A&F University, Xianyang, 712100 China; 3 National Saline-tolerant Rice Technology Innovation Center, South China, Zhanjiang, 524008 China; 4 Shenzhen Institute of Guangdong Ocean University, Shenzhen, 518108 China

**Keywords:** NaCl pre-treatment, Performance index, Rice, Electron transport efficiency

## Abstract

**Background:**

Salinity stress is an environmental constraint that normally develops concurrently under field conditions, resulting in drastic limitation of rice plant growth and grain productivity. The objective of this study was to explore the alleviating effects of NaCl pre-treatment on rice seedlings as well as the salt tolerance mechanisms by evaluating morph-physiological traits.

**Results:**

Variety Huanghuazhan, either soaked in distilled water or 25 mg/L Prohexadione calcium (Pro-Ca), were first hardened with varying concentrations of NaCl solutions (0 and 50 mM NaCl), and then subjected to varying degrees of salt stress (0 and 100 mM NaCl), indicated by S0, S1, S2 and S3, respectively. Growth analysis suggested that NaCl-pretreatment improved the root/shoot ratio in water-soaked rice plant at DAP 0. Data related to the reaction center density, photosynthetic electron transport efficiency, trapping efficiency were compared before (CK) using performance Index (PIabs). Compared to S2 (Pro-Ca-S2) treatment, PIabs did not show any difference with plants pre-treated with NaCl (S3 or Pro-Ca-S3). Rather than PIabs, significant difference was found in photosynthetic electron transport efficiency (Ψ_Eo_). The Ψ_Eo_ value in Pro-S2 was significantly lowered as compared to Pro-S3 treatment at DAP 7, and the decrease rate was about 6.5%. Correlation analysis indicated leaf PIabs was weak correlated with plant biomass while the quantum yield for reduction of the PSI end electron acceptors, trapped energy flux per reaction center and PSII antenna size displayed strong positive correlation with biomass. Additional analysis revealed that 100 mM NaCl significantly reduced leaf linear electron flux under low-light conditions, regardless of whether seedlings had been pre-treated with 50 mM NaCl or not.

**Conclusions:**

NaCl-induced salt tolerance was related to the robust photosynthetic machinery.

## Background

The global population will further expand to 11.2 billion by 2100, thereby needing 38% increase in food production during the following 30 years [1]. However, the increasing demand for food is being hampered by limited arable land and volatile environmental conditions. Salinization is a major environmental issue and currently about 20% of the world arable land and 33% of irrigated croplands are exposed to soil salinity [2]. To enhance productivity in saline conditions, it is essential to choose an appropriate method for mitigating salt stress and gain a deep understanding of the underlying mechanisms.

Rice (*Oryza sativa* L.) is a staple food crop for more than half of the global population [3] and is considered extremely sensitive to soil salinity [4]. Therefore, improving the capacity of rice to tolerate salinity are essential for global food security. Developing new rice germplasms that can tolerate salt (refer as inherited salinity tolerance) is certainly the most direct approach. As salinity stress tolerance is a polygenic trait, and most of the genes displayed trait specificity and growth stage specificity, there has been limited progress for increased salinity tolerance [5]. Another approach is agronomical practices such as the pre-treatment with NaCl to induced salinity tolerance [6, 7] or plant growth-regulating substances [8–10]. These practices can trigger plants to activate various mechanisms in response to salt stress, for example, antioxidant enzyme, ion homeostasis as well as hormone activation [11]. The previous evidence demonstrated that an improved understanding in induced salinity tolerance would be helpful to unravel the key components of the plant salt tolerance network. Importantly, these stress-reduction pre-treatment approaches are typically inexpensive and simple [12].

Rice cultivation usually adopted transplanted system in China [13]. In this cultivation system, the strong seedlings grown in nursery were transplanted to the field [14]. When transplanted into saline soil, rice plants are prone to experience salt shock event. To adapt to transplanting environmental conditions, salt tolerance of nursery seedling must be improved.

The benefit impact of NaCl-pretreatment on photosynthesis have been studied by several researchers [15, 16]. Photosynthesis consists of two basic processes [17], the ‘light reactions’ process involved in converting light energy into chemical energy, and the latter ‘carbon reactions’ process involved in the biochemical fixture of atmospheric CO_2_. However, most of these studies have used a large number of photosynthesis-related parameters involved in ‘carbon reactions’ process, for example, net photosynthetic rate; hence the conclusions drawn might be process specific. OJIP (defined by O, J, I, and P steps during a rapid polyphasic fluorescence rise) transient is a good approach to study the ‘light reaction’ process. Different with the determination of electron transport capacity through pulse amplitude modulation (PAM) technology, OJIP measurement can probe the electron fluxes between different components upstream, inside and downstream of photosystem II [18, 19]. Performance Index (PIabs) is a multiparametric expression of three independent steps, namely absorption of light energy (ABS), trapping of excitation energy (TR), and conversion of excitation energy to electron transport (ET) [20]. Any changes in the above processes would result in a change of PIabs [21]. For example, a limitation of electron transfer beyond Q_A_ was always found under stressful condition [22, 23], which in turn decrease the value of PIabs. However, in rice plants, it is unclear whether PIabs can be regarded as an indicator of NaCl-induced salt tolerance, and which components of PIabs showed responses.

In the field condition, the majority of leaves are exposed to rapidly changing light throughout the day. The ability to use rapidly increased light energy during light fluctuations determined light conversion efficiency and crop biomass [24]. Such robustness measurement for photosynthesis has gained attention, however, these measurements are usually taken under strictly controlled condition. MultispeQ, an open-source scientific instrument, providing a deeper understanding of the complex interactions between plants and their real environment [25]. Importantly, real-time environment factors (temperature, light intensity, humidity, location) and plant photosynthetic phenotypes are outputted.

Previous reports evaluated the physiological response in electron transport capacity through pulse amplitude modulation (PAM) technology to study the mechanism of salt stress [25, 26]. However, fewer reports have characterized the damage cause and activity of the electron transport chain in the photosynthetic apparatus, especially in the study of NaCl-induced salinity tolerance. In order to deeply investigate the photosynthetic adaptation mechanisms under salt stress, we studied pretreated NaCl effect on chlorophyll synthesis, PSII activity, and light potential of rice seedlings. We hypothesized that (1) salt-pretreatment improved plant vitality; (2) salt-pretreatment improved the response to light fluctuation; (3) salt-pretreatment change plant morphology; (4) Improved photosynthetic performance will ultimately favor increased biomass.

## Methods

### Plant material

In this study, the rice variety Huanghuazhan (HHZ) that is widely cultivated in southern China was used as plant material. For HHZ, its genomic information has been revealed [27]. This variety was provided by the College of Coastal Agriculture, Guangdong Ocean University.

### Growth condition and salt treatment

Experiment was conducted at Guangdong Ocean University from December 2022 to January 2023. To obtain uniform rice seedlings for experiment, healthy and uniform seeds were sorted manually and sterilize with 10% H_2_O_2_ solution for 10 min, then thoroughly rinse with distilled water [28], soaked in distilled water or 25 mg/L Pro-Ca for 28 h, and incubated at 30 °C for 5 days.

**Table:** 

Treatment	Description
S0	No salt stress for water-soaked plant during experiment
S1	50 mM NaCl pretreatment + 0 mM NaCl treatment
S2	0 mM NaCl pretreatment + 100 mM NaCl treatment
S3	50 mM NaCl pretreatment + 100 mM NaCl treatment
Pro-S0	No salt stress for Pro-Ca-soaked plant during experiment
Pro-S1	Pro-Ca-soaking + 50 mM NaCl pretreatment + 0 mM NaCl treatment
Pro-S2	Pro-Ca-soaking + 0 mM NaCl pretreatment + 100 mM NaCl treatment
Pro-S3	Pro-Ca-soaking + 50 mM NaCl pretreatment + 100 mM NaCl treatment

The small seedlings were then transferred to a 96-well Generic Hydroponic Plant Grow System and the plants were irrigated with 1/2 Hoagland nutrient solution [29]. These seedlings were cultured in a growth chamber under the day/ night temperature 28/25°C with the provision of 12 h light ((~ 4500 lx light intensity) and 12 h dark [30]. The relative humidity was maintained at 75% throughout the experiment. In the practice, rice transplanting generally occurred when rice seedlings had three leaves [31]. In our experiment, the nursery seedlings need to undergo NaCl-pretreatment before third-leaf stage. During the second-leaf stage, seedlings were exposed to 50 mM NaCl for 2 days. Then the plants were placed in a Hoagland solution containing 100 mM NaCl for 7 days. Previously, 50 mM NaCl was thought as sublethal dose for rice seedlings while 100 mM NaCl was lethal dose [6]. To sum up, the experiment includes 4 groups for water soaked or Pro-Ca-soaked plant.

### Chlorophyll (chl) *a* fluorescence transient

Rice plants were dark adapted overnight. The most fully expanded leaf (third leaf) was placed in the PAM-2500 fluorometer’s leaf clip (Heinz Walz, Effeltrich, Germany). Measurements were carried out with 6 or 8 replicates (two plants were selected per container) at DAP (days after NaCl pretreatment) 2 and 7. A red saturating light pulse of 3000 µmol photons m^− 2^ s^− 1^ was used to trigger OJIP transients during the measurement. The fluorescence transient characterized by the O (all RCs open, measured at about 50 µs), J (2 ms), I (30 ms), and P (F_m_) steps, correspond to the redox states of the photosystems [32]. JIP test was conducted to analyze the transient and detailed parameter explanation can be seen in Table 1. Performance Index on an absorption basis, PIabs, was calculated to evaluate the vitality of a plant (Eq. 1).

**Table 1:** JIP-test parameters for the analysis of the Chl a fluorescence transient

Parameters	Formulae	Definition
Fo	F_50µs_	Minimum fluorescence intensity at 50 µs
Fm	F_P_	Maximal fluorescence intensity
F_J_	F_2ms_	Fluorescence intensity at the J step (2 ms)
F_I_	F_30ms_	Fluorescence intensity at the I step (30 ms)
V_J_	V_j_= (F_j_-Fo)/(Fm-Fo)	Relative variable fluorescence at 2 ms
V_I_	V_i_= (F_i_-Fo)/(Fm-Fo)	Relative variable fluorescence at 30 ms
Ψ_Eo_	Ψ_Eo_ = ETo/TR = 1-V_j_	Probability that a trapped exciton moves an electron into the electron transport chain beyond Q_A_-
Mo	Mo = 4(F_300µs_−F_50µs_)/(Fm − F_50µs_)	The initial slope of the transient
φPo	φPo= [1-(Fo/Fm)] = Fv/Fm	The maximum quantum yield for primary photochemistry
φEo	φEo= [1-(Fo/Fm)]*(1-V_j_)	Quantum yield for electron transport
φRo	φRo= [1-(Fo/Fm)]*(1-V_i_)	Quantum yield for reduction of the PSI end electron acceptors: Ferredoxin and NADP
ABS/RC	ABS/RC = Mo*(1/ V_j_)*(1/φ_Po_)	Ratio of antenna chlorophyll to PSII reaction center chlorophyll (indicator of antenna size for PSII)
TRo/RC	TRo/RC = Mo*(1/ V_j_)	Trapped energy flux per reaction center
ETo/RC	ETo/RC = Mo*(1/ V_j_)* Ψ_Eo_	Electron transport flux per reaction center
REo/RC	REo/RC = Mo*(1/ V_j_)* Ψ_Eo_ *φ_Ro_	Reduction per reaction center: PSI end electron acceptors
DIo/RC	DIo/RC= (ABS/RC)- (TRo/RC)	Dissipated energy flux per reaction center
PI_abs_	PIabs=(RC/ABS)*[φPo/(1-φPo)]*[ψ_Eo_/(1-ψEo)]	Performance index on absorption basis. Multi-parameter photosynthetic performance index expressing energy conservation from absorption of light by antenna complexes of photosystem II to electron transport to intersystem electron acceptors

1 $$PI = \left[ {\frac{{RC}}{{ABS}}} \right]\left[ {\frac{{\Phi Po}}{{1 - \Phi Po}}} \right]\left[ {\frac{{\Psi {\text{Eo}}}}{{1 - \Psi {\text{Eo}}}}} \right]$$

### Light potential

Light potential (LP), which is defined as the capacity of linear electron flow to respond to sudden changes in light intensity [24]. Hand-held MultispeQ 2.0 instruments (https://photosynq.com) were used to take measurements at DAP 7. Water-soaked seedlings were moved to an artificial greenhouse for 1 h of acclimation (~ 50 mol m^-2^s^-1^). The dynamic response of PSII quantum efficiency (Φ_II_) and linear electron flow (LEF) was evaluated by the LP protocol (ambient, high PAR and then dark) after small modification [24]. The full protocol (https://photosynq.org/projects/rapid-ps-responses- acclimation dmk-zuo) required more than 60 s, and most researchers could not steadily clamp a leaf in the instrument. To avoid this case, the instrument is attached to a triangular bracket during measurement.

### Plant growth and chlorophyll content

At DAP 7, plants were harvested, root and shoot fresh weight (FW) (g/plant) were determined. The leaf pigments were extracted with 96% ethanol and absorbance at 663 (A_663_) and 645 (A_645_) nm were measured to determine chlorophyll content [33].

### Statistical analysis

Water soaking treatment was performed using four replicates (four containers), whereas Pro-Ca soaking treatment was performed using three replicates. All data are described as mean value ± SE (Standard Error). Statistical analyses were carried out with the SPSS 23.0 software (SPSS Inc., Chicago, USA), and graphs were created with the Origin 2021 software (Northampton, USA). The effects of NaCl pre-treatment on plant salt tolerance were investigated using one-way ANOVA. Duncan’s multiple range test was used to compare mean values at 0.05 or 0.1 probability levels.

## Results

### Growth

**Fig. 1:**
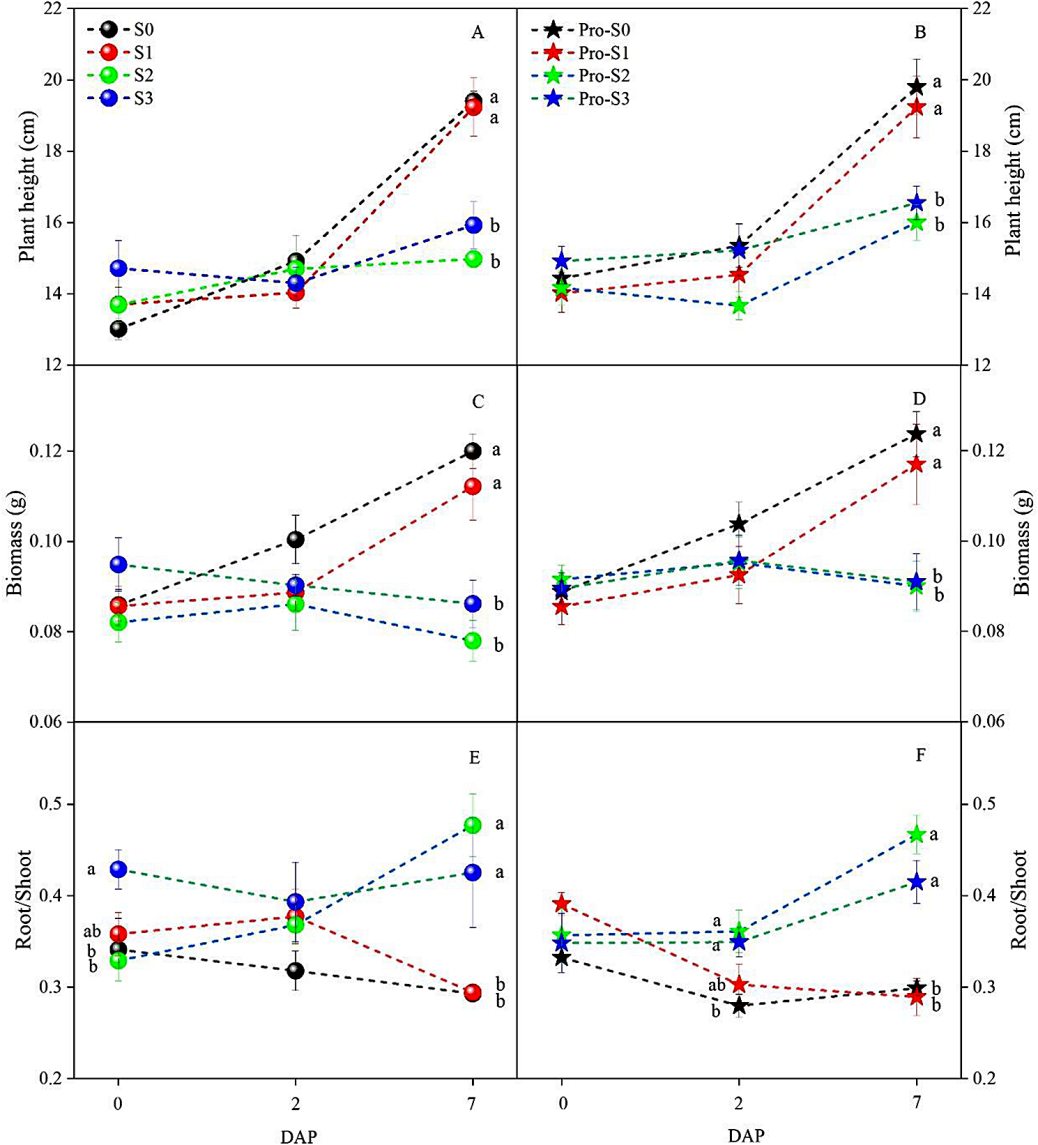
Effect of NaCl or Pro-Ca-NaCl pretreatment on plant height **(A, B)**, biomass **(C, D)** and root/shoot **(E, F)**

Salinity stress reduced plant growth and development traits. 100 mM NaCl showed significant influence on plant height and biomass (Fig. 1A, B, C, D). Using growth parameter as evaluation criteria, no difference was found between S2 and S3 treatments (Pro-S2 vs. Pro-S3) (Fig. 1). However, an increase in root/shoot (R/S) was observed in S3 treatment at DAP 0 (*P* < 0.05), and was about 20.7% higher than S0 and 23.3% higher than S2, respectively (Fig. 1E). The application of Pro-Ca affected the response of R/S. Interestingly, differences of R/S were detected at DAP 2 for Pro-Ca-soaked plant (Fig. 1F). Pro-S0 showed the lowest R/S value (*P* < 0.05), and was approximately 22.5% lower than Pro-S2 and 19.9% lower than Pro-S3, respectively.

### Chlorophyll content and light potential

**Fig. 2:**
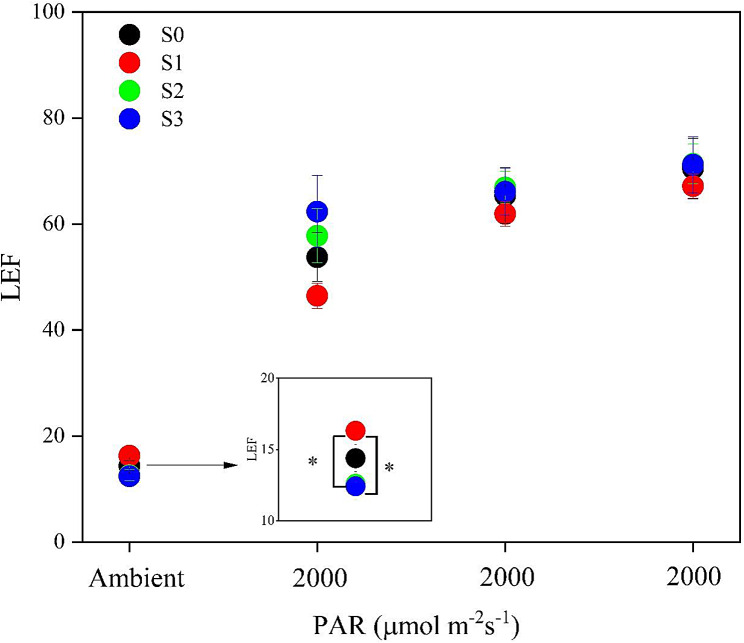
Effect of NaCl pretreatment on light potential. * indicated the difference at 0.05 possibility level

Chlorophyll content (Chl) (mg/g) was significantly (*P* < 0.05) decreased in S3 treatment, and decreased by 14.4% and 15.4% as compared to S1 and S2 treatments, respectively (Table 2). However, difference was not observed in Pro-Ca-soaked groups (Fig. 2B), meaning the application of Pro-Ca-soaked decreased the sensitivity of Chl to salt stress.

**Table 2:** Effect of NaCl or Pro-Ca-NaCl pretreatment on chlorophyll content at DAP 7

Treatment	Chlorophyll content (mg/g)	Chla/Chlb
S0	1.90 ± 0.10 a	2.94 ± 0.06 c
S1	1.92 ± 0.03 a	2.98 ± 0.03 bc
S2	1.71 ± 0.10 ab	3.13 ± 0.05 ab
S3	1.63 ± 0.04 b	3.21 ± 0.06 a
Pro-S0	1.98 ± 0.13 a	2.91 ± 0.09 a
Pro-S1	1.88 ± 0.02 a	2.98 ± 0.01 a
Pro-S2	1.77 ± 0.03 a	3.10 ± 0.06 a
Pro-S3	1.91 ± 0.07 a	3.04 ± 0.05 a

Under ambient low light conditions, the S1 treatment had the highest LEF, which was significantly higher than that in the S2 and S3 treatments **(**Fig. 2**)**. With the exposure to full sunlight i.e. 2000 µmol photons m^− 2^ s^− 1^, the LEF value increased accordingly. Notably, plants in S3 treatment showed 34.2% increase in initial slope (*P* > 0.05) compared to S1 treatment. It indicated a greater photochemistry efficiency in response to light fluctuation.

### Chlorophyll a fluorescence transient analysis

**Fig. 3:**
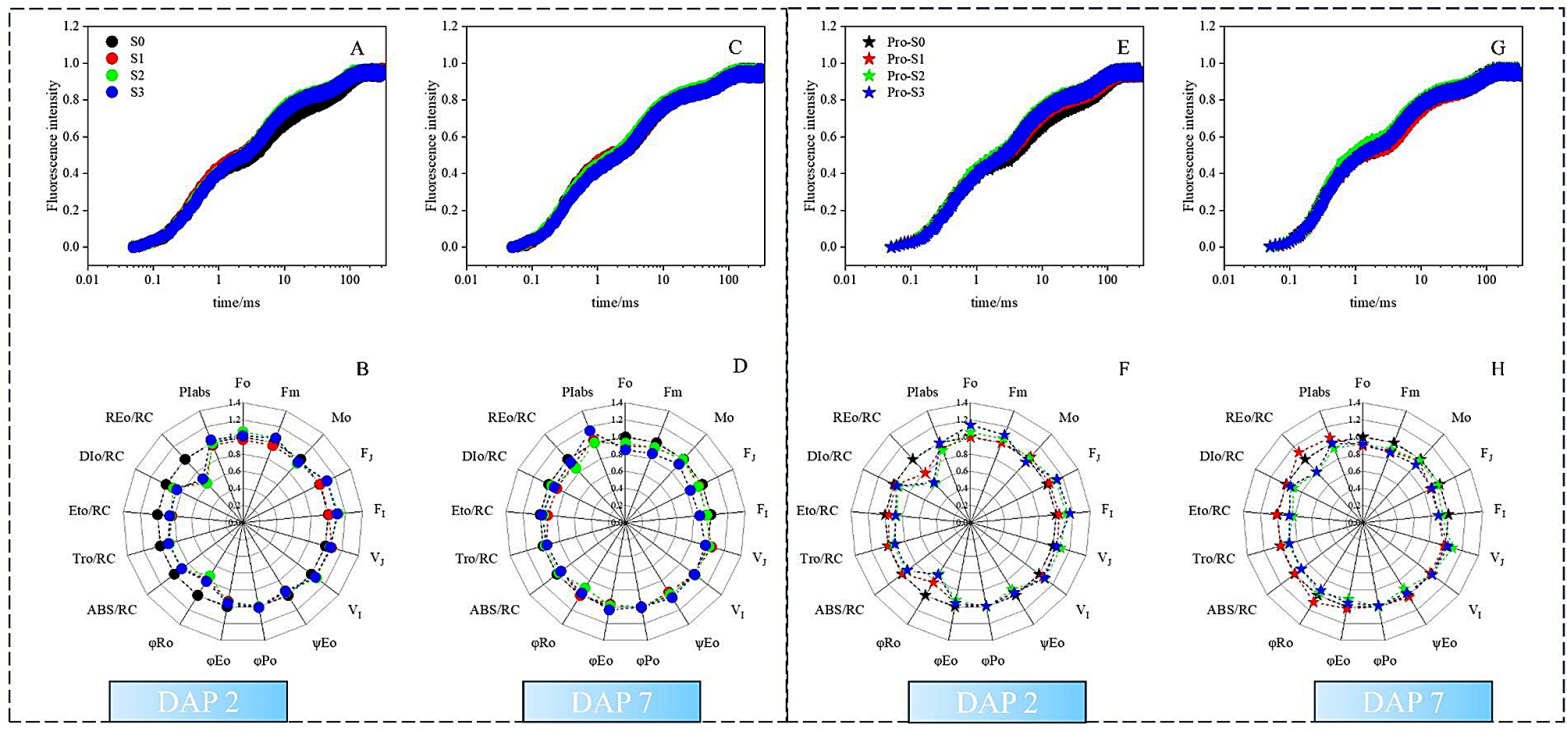
Effect of NaCl **(A, B, C, D)** or Pro-Ca-NaCl **(E, F, G, H)** pretreatment on O-J-I-P fluorescence transient and JIP parameters at DAP 2 and 7

The kinetic curves of fast chlorophyll fluorescence induction were evaluated to assess the impact of various salt treatments on the photosynthesis system (PS) activity of rice leaves. Clearly, all treatments had a typical polyphasic O-J-I-P shape (Fig. 3); however, considerable changes occurred for the transient shape under different salt treatments.

The F_v_/F_m_ and PIabs are two widely used fluorescence parameter to describe plant physiological state in stress-related studies. Although both parameters are relative stable and showed no difference during the experiment (Table 3), plants in S2 or ProS2 had lowest PIabs value (Fig. 3). PIabs is a consolidated parameter composed of three individual components: active RC density on a Chl basis (RC/ABS), trapping probability (P_TR_), and electron transport probability (PET). Interestingly, we detected differences in the probability that a trapped exciton moves an electron into the electron transport chain beyond Q_A_^−^ (Ψ_Eo_) (Table 3). On the one hand, Ψ_Eo_ in S3 treatment had the maximum value (0.51) at DAP 7, displaying significantly difference with that in S1 treatment (*P* < 0.05); On the other hand, Ψ_Eo_ in Pro-S2 treatment was significantly lowered than that in Pro-S3 treatment at DAP 7 (Fig. 3). The combined results in water-soaked and Pro-Ca-soaked plants showed the beneficial effect of hardening techniques on electron transfer efficiency. Even though a significant difference was found in RC/ABS in Pro-Ca-soaked plant at DAP 7 (*P* < 0.05), but not in water-soaked plant, no difference was observed between Pro-S2 and Pro-S3 (Table 3).

**Table 3:** The significance level for JIP parameters in water-soaked and Pro-Ca-soaked treatments

*P* value	Day 2		Day 7	
	Water-soaked	Pro-Ca-soaked	Water-soaked	Pro-Ca-soaked
V_J_	< 0.05	0.059	< 0.05	< 0.05
V_I_	< 0.05	< 0.05 05	0.084	< 0.05
ѱEo	< 0.05	0.059	< 0.05	< 0.05
Mo	0.890	0.774	0.532	0.309
φPo	0.421	0.582	0.263	0.093
φEo	< 0.05	< 0.05	0.119	< 0.05
φRo	< 0.05	< 0.05	0.085	< 0.05
ABS/RC	0.300	0.501	0.728	< 0.05
TRo/RC	0.346	0.424	0.833	< 0.05
ETo/RC	0.061	< 0.05	0.656	< 0.05
DIo/RC	0.182	0.830	0.263	0.179
REo/RC	< 0.05	< 0.05	0.371	< 0.05
PIabs	0.910	0.672	0.210	0.137

### Correlation between JIP parameters and biomass

**Fig. 4:**
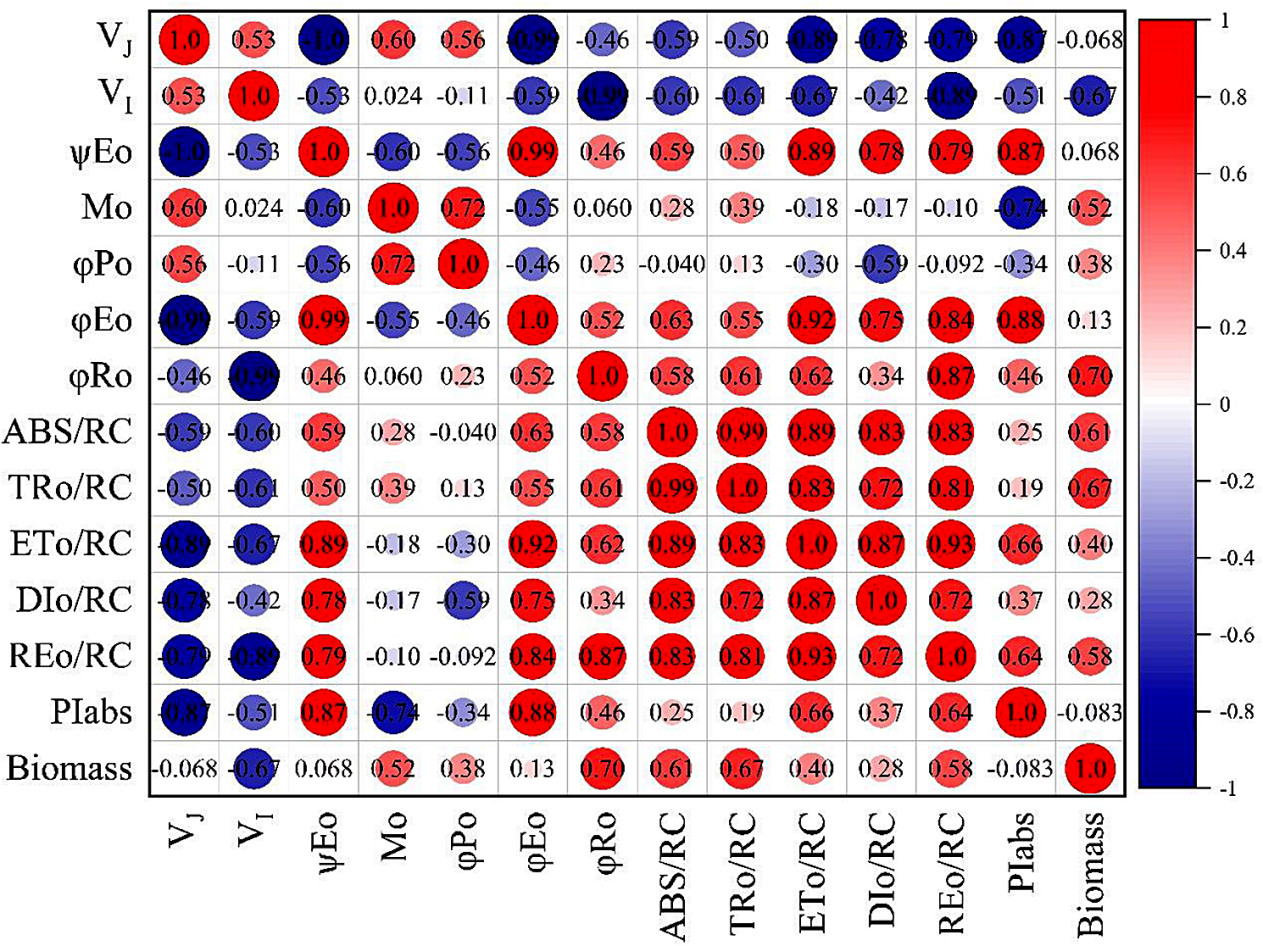
Correlation between JIP parameters and biomass

Figure 4 showed the correlations of the JIP parameters and biomass at DAP 7. PIabs showed negative correlation (Pearson correlation coefficient=-0.083) with biomass, meaning photosynthetic adaptive capacity was not related to growth under salt stress condition. Among these JIP parameters, the quantum yield for reduction of the PSI end electron acceptors (φRo), trapped energy flux per reaction center (TRo/RC) and PSII antenna size (ABS/RC) displayed strong positive correlation with biomass.

## Discussion

Improving the performance of transplanted rice under saline conditions is critical to rice production [34]. The seedling hardening technique has emerged as an effective and practical method for increasing salt tolerance and decreasing the inhibitory effect of stress on crop growth and development. The present work investigated the benefit effect of hardening technique either alone or in combination with plant growth regulator Pro-Ca on rice plants, aimed to draw the whole picture of the mechanisms for the acquired salt tolerance.

### NaCl-pretreatment improved electron transport efficiency

The photosynthetic electron transfer chain (ETC) enable the conversion of light energy into chemical energy [17]. OJIP analysis describes a set of photochemical parameters related to both photosystems I and II, and provides detailed information about the electron transfer process. The maximum quantum yield of PSII(F_v_/F_m_) derived from fluorescence transient has been widely used to identify tolerance to environmental stress [35–37]. Our data showed that under severe NaCl condition, rice plant growth was suppressed (Fig. 1, D); however, the F_v_/F_m_ ratio remained constant (~ 0.8) (Fig. 3). This suggested rice plants able to maintain a high efficiency of PSII in stress conditions [38]. Previous reports indicated PIabs showed advantage than F_v_/F_m_ in detecting stress response [39, 40]. F_v_/F_m_ is related to the maximal (F_m_) and minimal (F_o_) fluorescence levels, whereas PIabs includes both the absolute value and the trajectory by which the fluorescence intensity reaches from F_o_ to F_m_. Our work did not find significant differences in PIabs among treatments (Fig. 3D, H); however, PIabs value in S3 or Pro-S3 treatment was always higher than that in S2 or Pro-S2 treatment.

Interestingly, a significant difference was found in the photosynthetic electron transport efficiency, which is one of the components used to calculate PIabs (Eq. 1). The O–J phase in Chl a fluorescence induction represented the photochemical phase, involving in Q_A_ reduction [41]. Relative variable fluorescence at J step (V_J_) in S2 and Pro-S2 was notably higher than in S3 and Pro-S3 treatments, respectively (Fig. 3G, *P* < 0.05), leading to a decreased probability that a trapped exciton moves an electron into the electron transport chain beyond Q_A_- (Ψ_Eo_) (*P* < 0.05). In other words, the acceptor side of PSII suffered greater damage from salt stress in S2/Pro-S2 as compared to S3/Pro-S3. The reason might be that the exposure of rice seedlings to a primary stress (NaCl-pretreatment) elicited a short-term stress memory and improved its adaptation when encountering a secondary salt stress [42]. Overall, our results clearly showed that the benefits of NaCl pretreatment were linked to the enhanced electron transport efficiency.

### NaCl-pretreatment did not improve the response to high light

Unlike fast fluorescence transients, pulsed amplitude modulation (PAM) measurements estimate the actual linear electron transport rate, allowing the salt sensitive and tolerant genotypes to be distinguished [43]. MultispeQ was created as an open-source multifunctional tool that can function as a fluorometer, chlorophyll meter, and bench-top spectrometer [44]. We only considered chlorophyll fluorescence function in this study due to the high sensitivity of absorbance-based measurements. Previous research found that salt stress reduced LEF [43, 45]. This was in agreement with our finding of LEF under low light conditions (Fig. 2), i.e. LEF in S2 and S3 treatments was significantly lower compared to the S1 treatment (*P* < 0.05). However, there was no significant difference in LEF under high light conditions. This indicated that both S2 and S3 treatments exhibited a faster initial response of LEF to full sunlight (2000 mol m^− 2^s^− 1^). This unveiled a previously unrecognized mechanism that salt stress enhanced the initial response of plant to high light. For rice cultivar HHZ, such effective strategy helps to prevent over-reduction of the electron transport chain, which produce toxic Reactive oxygen species (ROS). However, further study needs to explore whether this phenomenon is dependent on salt dosage or variety. Since no significant difference in LEF performance between S2 and S3 treatment, NaCl-pretreatment did not improve the response to high light.

### NaCl-pretreatment increased root/shoot ratio

Plant water supply is thought to be the most important factor in crop salt tolerance [46]. As a result, the development of a resource-acquiring organ root is critical to a seedling’s ultimate success. Although shoots are thought to be more sensitive to salinity than roots [47], new understandings is beginning to emerge [48]. The root/shoot ratio, an important anti-stress indicator at the plant level, which conveyed information about the relative sensitivity of root and shoot growth to stress. The current study identified that R/S response in hardened plants (Fig. 1E). Is a higher root/shoot ratio a good predictor for salt tolerance? This question is seldom discussed. From the mechanism point of view, osmotic effect rather than salt-specific effect causes root/shoot change. In the short term, an increase in root/shoot ratio is an adaptive response, and a larger root system may favor the retention of toxic ions [49]. The increased root growth aids in the development of salt tolerance associated with moisture and nutrition absorption [50]. However, such a change may reduce the shoot’s ability to supply photo-assimilates to the roots, affecting plant growth and survival in the long run [51]. Our findings were consistent with previous studies that yield (biomass) and R/S showed negative relationship at different salinity levels in tomato [52]. Therefore, an increase of R/S did not contribute to increase crop productivity in the long term.

### PIabs is not related to growth under salt stress condition

In the present study, plants responded to salinity with two ways: through preexisting resistance mechanisms (S2 or Pro-S2) and the acquired tolerance (S3 or Pro-S3). A key question is whether an improved photosynthetic activity under salt stress condition meant an increased rice biomass? Interestingly, a negative correlation was found between biomass and PIabs (Fig. 4). The results suggested that PIabs was not a good predictor of plant growth under salt stress condition. Photosynthetic characteristics measured by fluorescence, e.g. ETR and F_v_/F_m_ coupled with crop growth in well-grown conditions [53]. Under stress condition, the response of these fluorescence parameters just indicate the activation of other metabolic pathways [53]. Most importantly, PIabs was related to the processes of ‘light reaction’, but not CO_2_ assimilation for biomass formation. Interestingly, correlation analysis in our study showed that the quantum yield for reduction of the PSI end electron acceptors (φRo), trapped energy flux per reaction center (TRo/RC) and PSII antenna size (ABS/RC) were the potential parameters to predict biomass. However, M Ashraf and PJ Harris [54] demonstrated that photosynthetic capacity was not a general indicator for salt tolerance. Furthermore, numerous indices, such as ion accumulation, photosynthetic pigments, and photosynthesis gas exchange, are necessary but not sufficient to define salt tolerance and predict crop growth [55]. Further investigation is required to verify these potential parameters and identify new parameter for predicting crop growth under salt stress.

## Conclusion

In this study, the photosynthetic mechanism underlying the acquired salt tolerance was evaluated through analysis of the morpho-physiological traits. An increased R/S was found in hardened plants for water-soaking treatment but not Pro-Ca treatment. Importantly, we found NaCl-pretreatment decreased PSII acceptor side limitation, as displayed by the increased probability that a trapped exciton moves an electron into the electron transport chain beyond Q_A_- (Ψ_Eo_). This mechanism decreased the risk of PSII photo-inhibition for pre-treated rice plants. Photosynthetic activity (PIabs) showed a weak correlation with rice biomass, and new parameters were needed for reliable prediction. While NaCl pretreatment did not increase the response of LEF to high light, such a phenomenon was found in salt-stressed plants, regardless of whether they were pretreated or not. Is this a resistance mechanism shared by stressed plants? Further investigations will bring more alternatives for crop salt tolerance mechanism.

## Data Availability

The datasets generated during the current study are available from the corresponding author on reasonable request.
